# Supplementary material for: Diagnostic Accuracy of Differential-Diagnosis Lists Generated by Generative Pretrained Transformer 3 Chatbot for Clinical Vignettes with Common Chief Complaints: A Pilot Study
Source: Int J Environ Res Public Health. 2023 Feb 15;20(4):3378. doi: 10.3390/ijerph20043378 (PMC9967747; doi:10.3390/ijerph20043378)
Supplement: Supplementary file 1 [file ijerph-20-03378-s001.zip › supplemental_material1.pdf]

Supplemental Figure S1: sample vignette, single correct diagnose, and five differential diagnoses

Chief complaints	Case
sore throat	ID: 23 year-old man<br>History of Present illness: He was in his usual state of health until 3 days prior when he developed sore throat and runny nose. He denied any other symptoms, such as cough, fever, headache, abdominal pain, nausea, vomiting, back pain, change in urine, or appetite loss.<br>Past Medical History: Past Medical History was insignificant.<br>Medication: Medications included only acetaminophen. He denied medication allergies<br>Social/Family History: He was a technical illustrator who had 3-4 beers each week. Family history was unremarkable.<br>Physical examination: The physical examination of his head, ears, eyes, nose, and throat (HEENT) shows congestion in his throat without white pus on his tonsils, but no other notable abnormalities including in lymph nodes swelling. The rest of the physical examination is also unremarkable.

Chief complaints	One Correct answer
sore throat	Upper respiratory tract infection

Chief complaints	Five differential diagnoses
sore throat	1. Upper respiratory tract infection<br>2. COVID-19<br>3. Influenzae<br>4. Pharyngitis<br>5. Allergic rhinitis
