# Supplementary material for: Diagnostic Accuracy of Differential-Diagnosis Lists Generated by Generative Pretrained Transformer 3 Chatbot for Clinical Vignettes with Common Chief Complaints: A Pilot Study
Source: Int J Environ Res Public Health. 2023 Feb 15;20(4):3378. doi: 10.3390/ijerph20043378 (PMC9967747; doi:10.3390/ijerph20043378)
Supplement: Supplementary file 1 [file ijerph-20-03378-s001.zip › supplemental_material2.pdf]

Supplemental Figure S2: Single correct diagnosis of the clinical vignettes and the ten differential-diagnosis lists created through ChatGPT-3.

No	Chief complaint	Correct diagnosis	Differential-diagnosis list by ChatGPT-3
1-1	Abdominal pain	Cholecystitis	1. Gastroesophageal reflux disease (GERD)<br>2. Peptic ulcer disease<br>3. Gastritis<br><u>4. Acute cholecystitis</u><br>5. Pancreatitis<br>6. Hiatal hernia<br>7. Pancreatic cancer<br>8. Small intestine obstruction<br>9. Appendicitis<br>10. Stomach cancer
1-2	Abdominal pain	Appendicitis	<u>1. Appendicitis</u><br>2. Gastroenteritis<br>3. Ovarian torsion<br>4. Urinary tract infection<br>5. Ectopic pregnancy<br>6. Pelvic inflammatory disease<br>7. Endometriosis<br>8. Constipation<br>9. Irritable bowel syndrome<br>10. Diverticulitis
1-3	Abdominal pain	Acute pancreatitis	<u>1. Acute pancreatitis</u><br>2. Gastroenteritis<br>3. Peptic ulcer disease<br>4. Gallbladder disease<br>5. Hepatitis<br>6. Esophageal reflux disease (GERD)<br>7. Alcoholic liver disease<br>8. Appendicitis<br>9. Constipation<br>10. Food poisoning
2-1	Fever	Pyelonephritis	<u>1. Urinary tract infection</u><br>2. Kidney stone

			3. Pneumonia<br>4. Sepsis<br>5. Influenza<br>6. Gastroenteritis<br>7. Malignancy<br>8. Drug reaction<br>9. Thyroiditis<br>10. Rheumatoid arthritis
2-2	Fever	Infectious mononucleosis	1. Acute tonsillitis<br>2. Acute pharyngitis<br>3. Mononucleosis<br>4. Influenza<br>5. Measles<br>6. Mumps<br>7. Streptococcal infection<br>8. Adenovirus infection<br>9. Parvovirus infection<br>10. HIV infection
2-3	Fever	Infective endocarditis (IE)	1. Infective endocarditis<br>2. Pyelonephritis<br>3. Pneumonia<br>4. Urinary tract infection<br>5. Acute bronchitis<br>6. Gastroenteritis<br>7. Sepsis<br>8. Influenza<br>9. Tuberculosis<br>10. Malaria
3-1	Chest pain	Myocardial infarction	1. Acute coronary syndrome (e.g.myocardial infarction, unstable angina)<br>2. Pulmonary embolism<br>3. Aortic dissection<br>4. Pneumonia<br>5. Esophageal spasm or acute gastroesophageal reflux disease (GERD)<br>6. Pericarditis

			7. Hypertensive crisis<br>8. Pulmonary hypertension<br>9. Anaphylaxis<br>10. Pneumothorax
3-2	Chest pain	Acute myocardial infarction (AMI)	1. Myocardial infarction (heart attack)<br>2. Angina pectoris<br>3. Aortic aneurysm<br>4. Pulmonary embolism<br>5. Pericarditis<br>6. Acute coronary syndrome<br>7. Cardiomyopathy<br>8. Aortic stenosis<br>9. Mitral stenosis<br>10. Pulmonary hypertension
3-3	Chest pain	Pulmonary embolism (PE)	1. Myocardial infarction (heart attack)<br>2. Aortic dissection<br>3. Pulmonary embolism<br>4. Pericarditis<br>5. Angina<br>6. Hypertrophic cardiomyopathy<br>7. Anterior wall myocardial infarction<br>8. Mitral valve prolapse<br>9. Panic attack<br>10. Pulmonary hypertension
4-1	Breathing difficulty	Pneumothorax	1. Pneumonia<br>2. Pulmonary embolism<br>3. Chronic obstructive pulmonary disease (COPD)<br>4. Asthma<br>5. Bronchitis<br>6. Collapsed lung (pneumothorax)<br>7. Pulmonary edema<br>8. Heart failure<br>9. Pleural effusion<br>10. Esophageal cancer
4-2	Breathing	Chronic	1. Chronic obstructive pulmonary disease

	difficulty	obstructive pulmonary disease (COPD)	(COPD)<br>2. Bronchitis<br>3. Emphysema<br>4. Pulmonary fibrosis<br>5. Chronic bronchitis<br>6. Asthma<br>7. Pulmonary embolism<br>8. Lung cancer<br>9. Pneumonia<br>10. Heart failure
4-3	Breathing difficulty	Hypersensitivity pneumonia	1. Pneumonia<br>2. Bronchitis<br>3. Asthma<br>4. Pulmonary embolism<br>5. Tuberculosis<br>6. Influenza (the flu)<br>7. Heart failure<br>8. Chronic obstructive pulmonary disease (COPD)<br>9. Allergic reactions<br>10. Anaphylaxis
5-1	Joint pain	Rheumatoid arthritis	1. Rheumatoid arthritis<br>2. Osteoarthritis<br>3. Gout<br>4. Psoriatic arthritis<br>5. Systemic lupus erythematosus<br>6. Scleroderma<br>7. Raynaud's phenomenon<br>8. Carpal tunnel syndrome<br>9. Tennis elbow (lateral epicondylitis)<br>10. De Quervain's tenosynovitis
5-2	Joint pain	Osteoarthritis	1. Osteoarthritis<br>2. Rheumatoid arthritis<br>3. Gout<br>4. Bursitis<br>5. Tendinitis

			6. Baker's cyst<br>7. Knee ligament injuries<br>8. Meniscal tears<br>9. Knee joint infections<br>10. Obesity
5-3	Joint pain	Systemic lupus erythematosus (SLE)	1. Scleroderma<br>2. Rheumatoid arthritis<br>3. Systemic lupus erythematosus (SLE)<br>4. Dermatomyositis<br>5. Sjögren's syndrome<br>6. Psoriasis<br>7. Sunburn<br>8. Erythema multiforme<br>9. Rosacea<br>10. Polyarteritis nodosa
6-1	Vomiting	Small bowel obstruction	1. Gastroenteritis (inflammation of the stomach and intestines, often caused by a viral or bacterial infection)<br>2. Peptic ulcer disease (ulcers in the lining of the stomach or small intestine)<br>3. Gastroparesis (delayed emptying of the stomach)<br>4. Gallbladder inflammation or infection (cholecystitis)<br>5. Pancreatitis (inflammation of the pancreas)<br>6. Small intestine obstruction<br>7. Appendicitis (inflammation of the appendix)<br>8. Diverticulitis (inflammation of small pouches in the intestine)<br>9. Constipation<br>10. Irritable bowel syndrome (a disorder that affects the large intestine)
6-2	Vomiting	Subarachnoid hemorrhage	1. Migraine headache<br>2. Cluster headache

			3. Tension-type headache<br>4. Acute sinusitis<br>5. Temporal arteritis<br>6. Hypertensive crisis<br>7. Subarachnoid hemorrhage<br>8. Reversible cerebral vasoconstriction syndrome<br>9. Intracranial hypotension<br>10. Spontaneous intracranial hypotension
6-3	Vomiting	Bowel obstruction	1. Acute gastroenteritis<br>2. Acute cholecystitis (inflammation of the gallbladder)<br>3. Acute pancreatitis<br>4. Small bowel obstruction<br>5. Mesenteric ischemia (restricted blood flow to the intestine)<br>6. Inflammatory bowel disease (such as Crohn's disease or ulcerative colitis)<br>7. Diverticulitis (inflammation of small, bulging sacs in the intestine)<br>8. Appendicitis<br>9. Abdominal aortic aneurysm<br>10. Abdominal abscess
7-1	Ataxia/difficulty walking	Wernicke encephalopathy	1. Alcoholic cerebellar degeneration<br>2. Wernicke-Korsakoff syndrome<br>3. Intoxication or withdrawal from alcohol or other substances<br>4. Infection, such as meningitis or encephalitis<br>5. Vitamin deficiency, such as thiamine deficiency<br>6. Stroke or transient ischemic attack<br>7. Multiple sclerosis<br>8. Brain tumor<br>9. Chronic alcoholism<br>10. Neurodegenerative disorders, such as

			Parkinson's disease or multiple system atrophy
7-2	Ataxia/difficulty walking	Cerebral infarction (Wallenberg syndrome)	1. Stroke<br>2. Transient ischemic attack (TIA)<br>3. Vertebrobasilar insufficiency<br>4. Intracerebral hemorrhage<br>5. Brainstem infarction<br>6. Multiple sclerosis<br>7. Brain tumor<br>8. Acoustic neuroma<br>9. Migraine<br>10. Alcoholic cerebellar degeneration
7-3	Ataxia/difficulty walking	Cerebral infarction	1. Stroke (cerebrovascular accident)<br>2. Transient ischemic attack<br>3. Vestibular disorder<br>4. Hypertensive crisis<br>5. Hypoglycemic episode (related to diabetes)<br>6. Cardiac arrhythmia<br>7. Hemorrhage or aneurysm<br>8. Migraine headache<br>9. Seizure<br>10. Infectious or inflammatory condition (such as meningitis or encephalitis)
8-1	Back pain	Nonspecific back pain (musculoskeletal pain)	1. Muscular strain or sprain<br>2. Lumbar strain<br>3. Lumbar herniated disc<br>4. Lumbar spinal stenosis<br>5. Lumbar degenerative disc disease<br>6. Osteoarthritis<br>7. Spondylolisthesis<br>8. Ankylosing spondylitis<br>9. Fibromyalgia<br>10. Psychological factors
8-2	Back pain	Aortic dissection	1. Pulmonary embolism<br>2. Pneumonia

			3. Aortic dissection<br>4. Heart attack<br>5. Hypertensive crisis<br>6. Spinal cord injury<br>7. Acute coronary syndrome<br>8. Pulmonary edema<br>9. Chronic obstructive pulmonary disease (COPD)<br>10. Ankylosing spondylitis
8-3	Back pain	Malignant pleural mesothelioma	1. Chronic bronchitis<br>2. Pneumonia<br>3. Pulmonary fibrosis<br>4. Lung cancer<br>5. Pleural effusion<br>6. Bronchiectasis<br>7. Tuberculosis<br>8. Pulmonary embolism<br>9. Chronic obstructive pulmonary disease (COPD)<br>10. Asthma
9-1	Cough	Lung cancer	1. Pneumonia<br>2. Chronic obstructive pulmonary disease (COPD)<br>3. Bronchitis<br>4. Tuberculosis<br>5. Lung cancer<br>6. Pulmonary embolism<br>7. Pulmonary fibrosis<br>8. Heart failure<br>9. Bronchiectasis<br>10. Asthma
9-2	Cough	Occupational asthma	1. Allergic bronchitis<br>2. Bronchitis<br>3. Sinusitis<br>4. Acid reflux: Gastroesophageal reflux disease (GERD)

			5. Postnasal drip<br>6. Upper respiratory infection<br>7. Bronchial asthma<br>8. Pneumonia<br>9. Chronic obstructive pulmonary disease (COPD)<br>10. Pulmonary embolism
9-3	Cough	Mycoplasma pneumonia	1. Pneumonia<br>2. Bronchitis<br>3. Tuberculosis<br>4. Influenza<br>5. Measles<br>6. Whooping cough<br>7. Mumps<br>8. Pertussis<br>9. Leptospirosis<br>10. Q fever
10-1	Dizziness	Benign paroxysmal positional vertigo	1. Benign paroxysmal positional vertigo (BPPV)<br>2. Vestibular neuritis<br>3. Meniere's disease<br>4. Hypotension<br>5. Anemia<br>6. Migraine<br>7. Orthostatic hypotension<br>8. Dehydration<br>9. Hypoglycemia<br>10. Cardiovascular issues
10-2	Dizziness	Benign Paroxysmal Positional Vertigo (BPPV)	1. Benign paroxysmal positional vertigo (BPPV)<br>2. Vestibular neuritis or labyrinthitis<br>3. Meniere's disease<br>4. Stroke or transient ischemic attack (TIA)<br>5. Cardiovascular problems, such as orthostatic hypotension or arrhythmias<br>6. Neurological problems, such as multiple

			sclerosis or brainstem compression<br>7. Migraine-associated vertigo<br>8. Drug-induced dizziness<br>9. Anemia or other blood disorders<br>10. Thyroid or other hormonal imbalance
10-3	Dizziness	Benign Paroxysmal Positional Vertigo (BPPV)	1. Benign paroxysmal positional vertigo (BPPV)<br>2. Labyrinthitis<br>3. Vestibular neuritis<br>4. Meniere's disease<br>5. Acoustic neuroma<br>6. Migraine<br>7. Multiple sclerosis<br>8. Stroke<br>9. Transient ischemic attack (TIA)<br>10. Chronic fatigue syndrome
